# Intratumoral Microbiota in Lung Cancer: Emerging Roles in TME Modulation and Immunotherapy Response

**DOI:** 10.3390/ijms27010255

**Published:** 2025-12-25

**Authors:** Xue Yang, Liyuan Yin, Zhuoying Tian, Qinghua Zhou

**Affiliations:** Lung Cancer Center/Lung Cancer Institute, West China Hospital, Sichuan University, Chengdu 610041, China

**Keywords:** lung cancer, intratumoral microbiota, immune checkpoint inhibitors, tumor microenvironment, immunotherapy resistance, microbial metabolites

## Abstract

Intratumoral microbiota, once considered passive bystanders, are now recognized as active modulators of the tumor immune microenvironment (TIME)—the complex network of immune cells, stromal components, and signaling molecules within tumors—and ultimately shape immunotherapy outcomes in lung cancer. This review aims to elucidate the exact roles of intratumoral microbiota in lung cancer immuno-therapy responses and the potential mechanism, offering novel perspectives for overcoming resistance. We conducted a narrative review of the literature using a PubMed and Web of Science search of articles written in English from inception to November 2025. We summarize current evidence on the characteristics of intratumoral microbiota in lung cancer and their associations with patient outcomes following immune checkpoint inhibitor (ICI) treatment. We discuss how intratumoral microbes, their metabolites, and extracellular vesicles influence and remodel TIME, thereby either promoting or counteracting ICI efficacy. Furthermore, we explore the potential of microbial signatures as predictive biomarkers and highlight microbiota-targeted strategies—including probiotics, engineered bacteria, and rational antibiotic use—to overcome resistance and enhance clinical benefits. Collectively, available data support intratumoral microbiota as crucial modulators and promising therapeutic targets in lung cancer, and decoding their multifaceted interactions may inform precision microbiota-targeting strategies to improve patient outcomes.

## 1. Introduction

Lung cancer remains the leading cause of cancer-related mortality worldwide. Non-small cell lung cancer (NSCLC), including lung adenocarcinoma (LUAD) and lung squamous cell carcinoma (LUSC), accounts for the majority of lung cancer cases [1]. Classical risk factors such as smoking, environmental and occupational exposure to carcinogens (e.g., radon, asbestos, diesel exhaust), air pollution, and chronic pulmonary diseases, together with host genetic susceptibility and aging, collectively drive lung cancer risk and profoundly shape the biological characteristics of the tumor [2,3].

In recent years, therapeutic strategies for lung cancer have evolved from conventional surgery, radiotherapy, and chemotherapy to include targeted therapies and immunotherapies. In particular, immune checkpoint inhibitors (ICIs) targeting PD-1/PD-L1 and CTLA-4 have become major pillars of systemic treatment for advanced NSCLC and are being increasingly applied in earlier stages of disease [1,4,5,6]. However, only a subset of patients derives durable clinical benefit, and primary or acquired resistance to immunotherapy remains a pressing clinical challenge [7]. These phenomena highlight the urgent need to identify new determinants of immunotherapy efficacy and modifiable regulatory factors, including extrinsic tumor factors such as the tumor immune microenvironment (TIME) and the microbiome [7].

The TIME plays a pivotal role in lung cancer initiation, progression, and therapeutic response. It is composed of innate and adaptive immune cells—including T cells, B cells, natural killer (NK) cells, dendritic cells (DCs), and tumor-associated macrophages (TAMs)—as well as stromal cells, extracellular matrix components, and soluble mediators [8,9]. TAMs can be categorized into a classically activated TAM1 (M1-like) phenotype with pro-inflammatory and anti-tumor functions, and an alternatively activated TAM2 (M2-like) phenotype that promotes immunosuppression, angiogenesis, and tissue remodeling [8]. In parallel, metabolic and physical features of the TIME—such as hypoxia, nutrient deprivation, and extracellular acidification—further shape immune cell function, tumor cell plasticity, and therapeutic sensitivity [8,9,10].

The lower respiratory tract was traditionally considered sterile; however, with the advent of high-throughput sequencing, it is now recognized that the airways and lung tissue harbor a low-biomass yet diverse pulmonary microbiota [11]. Under homeostatic conditions, the respiratory microbiota contributes to maintaining epithelial barrier integrity, modulating mucosal immunity, and preventing colonization of pathogenic microorganisms, thereby supporting pulmonary immune tolerance and controlled inflammatory responses [12,13]. Conversely, respiratory dysbiosis—characterized by alterations in microbial diversity and composition, enrichment of pathobionts, loss of beneficial commensals, and changes in the metabolic milieu—is associated with various inflammatory airway diseases and with lung cancer progression [11]. These effects are increasingly regarded as important factors influencing lung carcinogenesis and therapeutic outcomes.

Microorganisms colonizing lung tumor tissues—collectively referred to as the intratumoral microbiota—have been progressively recognized as integral components and active modulators of the TIME [11]. Bacteria, fungi, and other microbes within the tumor can interact with pattern recognition receptors through microbe-associated molecular patterns, directly influencing tumor cells and infiltrating immune cells, thereby regulating cytokine and chemokine networks, and the infiltration and polarization of immune cells [14,15,16,17,18]. In addition, intratumoral microbes participate in metabolic reprogramming within the tumor microenvironment (TME). Their metabolites induce multiple oncogenic signaling pathways, epigenetic remodeling, and genetic alterations, thereby driving malignant transformation of pulmonary cells and tumor progression. Short-chain fatty acids (SCFAs), tryptophan, lactate, and their downstream metabolites can activate various signaling cascades, including NF-κB and MAPK pathways [19,20,21]. Moreover, certain microbial metabolites function as epigenetic regulators by modulating histone lactylation and acetylation, reshaping chromatin accessibility and transcriptional programs, and ultimately altering gene expression profile [20,22,23]. DNA damage induced by chronic inflammation and metabolite may promote the accumulation of genetic mutations and genomic instability [24], providing additional mechanistic links between dysbiosis and lung carcinogenesis. Conversely, the distinctive features of the lung TME and host-related factors may selectively enrich specific microbial communities [25,26,27], establishing a bidirectional feedback loop between the intratumoral microbiota and the TIME.

However, current evidence on the intratumoral microbiota in lung cancer remains fragmented, with contradictory findings regarding the tumor-promoting versus tumor-suppressive roles of specific microbes. A cohesive framework delineating how these microbes, through mechanisms, shape the TIME and influence ICI efficacy is still lacking. Therefore, this review aims to systematically integrate the latest evidence on the intratumoral microbiota in lung cancer and its crosstalk with the TIME, with a particular emphasis on its association with responses to immunotherapy. Specifically, we aim to: (1) summarize the composition and potential origins of the intratumoral microbiota in lung cancer; (2) elucidate the mechanisms by which these microbes and their metabolites modulate the TIME and ICI response; and (3) discuss microbiota-targeted strategies as adjunctive approaches to overcome therapeutic resistance and improve clinical outcomes.

## 2. Search Method

This work is a narrative review. Our aim was to comprehensively summarize recent advances regarding intratumoral and respiratory microbiota in lung cancer, their roles in modulating the tumor immune microenvironment, and their impact on responses to immunotherapy.

The core search terms and their combinations included: “lung cancer” OR “non-small cell lung cancer” OR “NSCLC”, together with “microbiota” OR “microbiome” OR “intratumoral microbiota” OR “tumor microbiome”, and “tumor microenvironment” OR “TME” OR “immune microenvironment”, and “immunotherapy” OR “immune checkpoint inhibitor” OR “PD-1” OR “PD-L1” OR “CTLA-4”. Additional records were identified by manually screening the reference lists of key original studies and review articles.

Inclusion criteria were: 1. Original experimental or clinical studies (in vitro, in vivo, or human studies) focusing on lung cancer; 2. Articles assessing intratumoral, lower respiratory tract microbiota in lung cancer; 3. Studies investigating the interaction between microbiota and the TIME and/or the efficacy, resistance, or biomarkers of cancer immunotherapy; 4. Review articles that provided mechanistic or translational insights relevant to the above topics; 5. Publications in English. Exclusion criteria were: 1. Articles not related to lung cancer or not involving microbiota; 2. Conference abstracts, editorials, comments, or letters without substantial original data or mechanistic discussion; 3. Case reports or very small case series without clear relevance to TME modulation or immunotherapy response; 4. non-English publications.

## 3. Cancer Immunotherapy in Lung Cancer

Lung cancer is one of the leading causes of cancer incidence and mortality worldwide; non-small cell lung cancer (NSCLC) comprises around 85% of cases, with small cell lung cancer (SCLC) accounting for the remaining 15%. Over the past decade, immunotherapy, particularly ICIs, has led to breakthrough advances in lung cancer therapy across all disease stages, significantly prolonging overall survival (OS) and reshaping the therapeutic landscape [1]. To date, the U.S. Food and Drug Administration (FDA) has approved three classes of ICIs targeting distinct immune checkpoints for cancer therapy, namely antibodies directed against cytotoxic T-lymphocyte–associated protein 4 (CTLA-4), programmed cell death protein 1 (PD-1), and programmed death-ligand 1 (PD-L1). Owing to their superior efficacy and safety profiles, anti-PD-1/PD-L1 antibodies are now used far more extensively than anti-CTLA-4 antibodies [4]. Additionally, vaccines, cytokines, adoptive cell transfer therapy, and oncolytic viruses have been integrated into the immunotherapeutic regimens for lung cancer [28].

A substantial body of clinical trial data has demonstrated the profound clinical value of ICIs in lung cancer, and these agents have been widely implemented in clinical practice. A systematic review and meta-analysis confirmed that neoadjuvant or perioperative immunotherapy improves pathological complete response, major pathological response, and event-free survival, with greater benefit observed in tumors exhibiting higher PD-L1 levels [29]. In patients with unresectable stage III NSCLC, the PACIFIC trial demonstrated that 1 year of consolidation therapy with durvalumab following concurrent chemoradiotherapy increased median progression-free survival (PFS) from approximately 6 months to 17 months and also significantly improved OS; consequently, consolidation durvalumab has become the global standard treatment for those patients [5]. In advanced NSCLC, PD-1/PD-L1 inhibitors (such as nivolumab, pembrolizumab, atezolizumab, and durvalumab) have demonstrated superiority over conventional chemotherapy in multiple pivotal phase III trials and are now established as standard first-line or subsequent-line regimens for patients without driver mutations [1]. In SCLC, immunotherapy has also conferred limited but clinically meaningful survival benefits. The IMpower133 and CASPIAN trials demonstrated that atezolizumab and durvalumab significantly prolong OS in patients with extensive-stage SCLC [6]. Beyond PD-1/PD-L1 inhibitors, the recently developed delta-like ligand 3-targeted bispecific T-cell engager tarlatamab has markedly improved OS in the second-line treatment of relapsed extensive-stage small cell lung cancer, as shown in the phase III DeLLphi-304 trial [30].

Despite these considerable advances, not all patients derive benefit from immunotherapy, and some even experience disease progression during ICI treatment [31]. The mechanisms of immunotherapy resistance remain incompletely understood and are currently thought to involve tumor-intrinsic factors, host-related factors, and TME–related factors. Tumor-intrinsic determinants include loss of tumor immunogenicity (such as low tumor mutational burden, depletion of neoantigens, specific gene mutations, and the IPRES transcriptional signature), antigen presentation deficiency (related to alterations in the interferon-γ signaling pathway, loss of heterozygosity of human leukocyte antigen, and loss or mutation of β2-microglobulin and transporter associated with antigen processing), dysregulation of signaling pathways (including MAPK, PI3K, WNT, and IFN pathways), and loss or downregulation of PD-L1 expression [7]. Host-related factors primarily include age, sex, smoking history, and composition of the gut microbiota. Importantly, the TME is considered the principal site in which resistance to immunotherapy develops. Within the TME, exhaustion or depletion of antitumor immune components (such as CD8^+^ and CD4^+^ T cells, B cells, and DCs), recruitment and expansion of immunosuppressive components (including regulatory T cells (Tregs), TAMs, myeloid-derived suppressor cells (MDSCs), cancer-associated fibroblasts (CAFs)), increased levels of immunosuppressive cytokines (such as TGF-α, TGF-β, VEGF, IL-6, and IL-8), and altered intratumoral metabolic profiles and hypoxia, collectively contribute to resistance to immunotherapy [7,32].

In recent years, beyond the gut microbiota, an increasing number of studies have revealed the presence of distinctive microbial communities within the TME itself, whose compositional and functional features are associated with the efficacy of ICI therapy. These intratumoral microbiota are thought to influence therapeutic response by modulating the immune microenvironment, reprogramming metabolic pathways, and reshaping epigenetic regulation.

## 4. Presence and Characteristics of Intratumoral Microbiota

**Figure 1:**
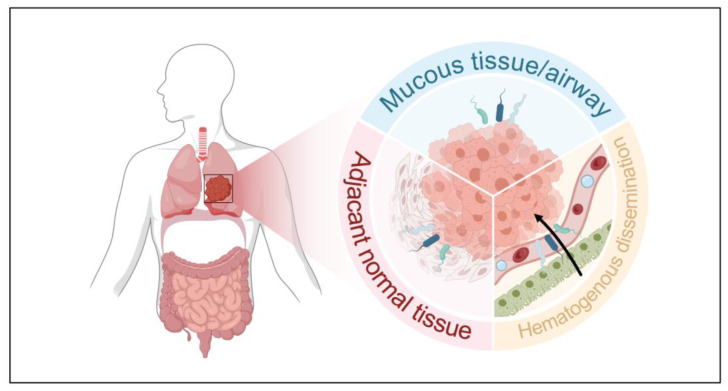
Potential sources for intratumoral microbiota in lung cancer. The figure depicts three hypothesized routes by which microbiota may enter the tumor microenvironment in lung cancer: (1) translocation across mucosal barriers, representing microbiota migration from the airway or alveolar mucosa into tumor tissue; (2) infiltration from adjacent normal tissue, where microbiota residing in nearby healthy lung tissue infiltrate along the expanding tumor margin; and (3) hematogenous dissemination, indicating migration of microbiota from other organs (e.g., digestive system) via the vascular system. The black arrow indicates the hematogenous dissemination of microbiota into the lung TME.

There are three potential sources for intratumoral microbiota in lung cancer: (1) translocation across mucosal barriers, (2) infiltration from adjacent normal tissue, and (3) hematogenous dissemination [33]. Gut-derived bacteria may also enter tumors via the circulatory system, reshaping the intratumoral community and immune microenvironment [34,35,36] (Figure 1). Various sampling methods have been employed to characterize the lung microbiome, including bronchoalveolar lavage fluid (BALF), bronchial brushing tissue, buccal sample, surgical resection tissue or exhaled breath condensate [37].

Microbial dysbiosis is a hallmark of lung cancer. In lung cancer patients, the pulmonary microbiome comprises bacteria (63.43%), eukaryotes (0.93%), viruses (0.57%), archaea (0.10%), and unclassified taxa (34.98%) [38]. With respect to bacteria, lung cancer is characterized by increased relative abundances of several phyla, including Actinobacteria, Bacteroidetes, and Proteobacteria [17]. The eukaryotic community is primarily composed of parasitic and fungal species [38]. As this review focuses on microbiota, we will mainly discuss the mycobiome rather than parasites. At the phylum level, the pulmonary fungi primarily consist of Ascomycota, Microsporidia, and Mucoromycota [38]. At the genus level, Blastomyces and Talaromyces are enriched in lung cancer group, whereas at the species level *Aspergillus sydowii* and *Talaromyces marneffei* are enriched [38,39,40]. Spatially, fungi are predominantly localized within macrophages in TIME [41]. With respect to viruses, current studies indicate that lung cancer rarely harbors viral species that are clearly tumor-specific or that differ significantly from those present in corresponding normal tissues [42]. In a subset of patients with lung cancer, Epstein–Barr virus (EBV) transcripts can occasionally be detected, and their presence has been associated with increased immune cell infiltration and elevated levels of immune checkpoint–related molecules [43]. Human papillomavirus (HPV) type 16 has also been detected in some lung cancer tissues; however, no associations have been demonstrated between HPV16 positivity and OS or features of the TIME [44]. Compared with adjacent non-tumorous tissues, tumor tissues harbor significantly higher microbial abundance and diversity, with a greater fungal burden in lung cancer tissues than in the corresponding normal tissues [41,45]. However, relative to normal lung tissue, lung cancer samples in other studies have been reported to exhibit decreased microbial diversity, increased bacterial burden, and alterations in dominant bacterial taxa [46,47]. In advanced lung cancer, enrichment of oral commensals such as *Streptococcus* and *Veillonella* has been observed, potentially activating IL-17, PI3K, MAPK, and ERK signaling pathways [48,49]. Microbial profiles also differ by lung cancer subtype. Intratumoral microbial α-diversity is higher in squamous cell carcinoma than in adenocarcinoma [17,25]. At the genus level, *Lactobacillus*, *Leptospira*, and *Mesorhizobium* are enriched in squamous carcinoma, whereas *Neisseria*, *Mycobacterium*, and *Bacteroides* are enriched in adenocarcinoma [17]. Age-dependent patterns have also been reported: the abundance of pathogenic bacteria (e.g., *Salmonella*, *Enterobacter*, *Enterococcus*) and opportunistic pathogens (e.g., *Cronobacter*, *Chlamydia*, *Streptococcus*, *Staphylococcus*) increases with age, while beneficial commensals decline [50,51]. In addition, certain species are associated with metastatic patterns; for example, *Finegoldia* abundance correlates negatively with pleural invasion, while *Renibacterium* is inversely associated with lymph node metastasis [52].

Microbial dysbiosis in lung cancer is caused by multiple factors. Besides the inhalation migration, ciliary clearance and cough reflex mentioned above, its composition of colonization is also regulated by the host’s genetic factors and immune microenvironment.

Host genetic background and local immune contexture are key determinants of the intratumoral microbiota in lung cancer. Multi-omics analyses indicate that somatic mutation profiles and transcriptional programs tightly co-vary with microbial community structure, with distinct histological subtypes and gene-expression patterns corresponding to characteristic microbial signatures [25]. Studies of familial lung cancer further support a role for inherited susceptibility in weakening epithelial barrier function and altering extracellular matrix and immune surveillance, thereby creating a permissive niche for opportunistic pathogens and depletion of beneficial commensals [53]. At the driver-mutation level, TP53 alterations and their co-mutation patterns not only affect epithelial barrier integrity and immune-response programs but are also associated with specific microbial configurations and differential responses to ICIs [54,55,56,57]. Apart from TP53, there is a lack of clear evidence that a single driver gene (such as EGFR, KRAS, ALK) directly determines the microbiota in lung cancer. Beyond genetic variation, the local immune contexture itself is a key ecological filter for the tumor-associated microbiome. Secretory IgA, T cell–derived cytokines and epithelial antimicrobial peptides (AMPs), together with neutrophil-mediated mechanisms, selectively opsonize, clear or contain specific taxa, thereby shaping which microbial lineages can persist within the tumor niche [26,27,58]. In lung cancer, multiple studies have documented tight associations between tumor/lung microbiota and γδT17 cells, cytotoxic CD8^+^ T cells, NK cells and myeloid-derived suppressor cells, and have shown that non–small cell lung cancers with distinct immune-cell compositions harbor different gut- and tumor-associated microbiota and display differential responses to ICIs [40,47,59,60,61,62,63,64]. Collectively, these data support a model in which host genetics and immune contexture do not simply “host” the intratumoral microbiome but form a tightly coupled network with it.

In turn, studies have shown that intratumoral microbial differences may contribute to variability in TIME and ICI response, forming a bidirectional regulatory loop. Tumoral microbial diversity is correlated with preferred immunotherapy efficacy [65,66], and enhanced survival benefit [67]. Responders to ICIs typically exhibit lower abundance of Proteobacteria and higher abundance of Bacteroidetes [66]. In patients with higher PD-L1 expression, lower airway microbiota shows greater similarity to the upper airway community [48]. Following immunotherapy, lung microbial diversity often declines, characterized by reductions in *Actinomyces*, Bacteroidetes, *Bifidobacterium* and *Prevotella*. In patients receiving greater clinical benefit from ICIs, such reduction is more significant [18,51,68].

## 5. Intratumoral Microbiota and Immunotherapy Response: Regulation of the Tumor Immune Microenvironment

Accumulating evidence indicates that the impact of intratumoral microbiota on the tumor immune microenvironment (TIME) is bidirectional, thereby eliciting divergent responses to immunotherapy (Table 1). On the one hand, intratumoral microbes reinforce antitumor features within the TIME. For example, an increased abundance of *Pasteurella* in the lung is associated with elevated CD8^+^ T-cell infiltration and reduced M2 macrophage polarization, ultimately restraining tumor growth [62]. On the other hand, intratumoral microbes also potentiate protumor features in the TIME. Specifically, increased abundance of *Riobacteriaceae* in lung has been linked to decreased CD8^+^ T cells and increased M2 macrophages, resulting in augmented tumor burden [62]. Mechanistically, intratumoral microbes regulate immune cells either directly or indirectly via metabolites or secreted factors (e.g., extracellular vesicles and AMPs).

### 5.1. Direct Regulation of the TIME by Intratumoral Microbiota

#### 5.1.1. Differential Effects of Intratumoral Microbes on Antigen-Presenting Cells (APCs)

*Talaromyces marneffei*, a fungal species, has been reported to be upregulated in the lung cancer microenvironment and is frequently localized within macrophages. At high concentrations, *T. marneffei* increases intramacrophage ARG-1 expression and promotes the arginine–ornithine cycle; concomitantly, the transcription factor STAT6—critical for M2 polarization—is upregulated. Collectively, these changes drive macrophage M2 polarization and increase PD-1 expression on the macrophage surface [38].

Conversely, aerosolized *Lactobacillus rhamnosus* can act directly on macrophages and DCs, upregulating costimulatory molecules (CD80/CD86) and MHC class II expression, thereby promoting the maturation of AMs, CD103^+^ DCs, and CD11b^+^ DCs. Moreover, *L. rhamnosus* downregulates M2 macrophage-associated genes (e.g., *Il10*, *Irf4*, *Ido*), reduces the expression of immunosuppressive cytokines *IL-10* and *TGF-β*, while increasing M1-associated markers (e.g., *IL-12*, *IRF5*). These effects suppress M2 polarization, promote M1 polarization, and enhance immune surveillance [72]. In addition, the pulmonary microbiota can upregulate CXCL9 in both cancer and myeloid cells, facilitating recruitment of CD8^+^ T cells into the tumor core and thereby improving the therapeutic efficacy of nivolumab [66].

#### 5.1.2. Remodeling of Innate Immune Cells by Intratumoral Microbiota

Enrichment of oral commensals such as *Veillonella parvula* in lung promotes neutrophil recruitment [48]. *Acidovorax temperans* has been shown to skew neutrophil maturation toward a protumor phenotype, characterized by upregulation of markers associated with excessive infiltration (Icam), immunosuppression (Cd274/PD-L1), and tumor promotion (Siglecf). These matured neutrophils secrete CSF1, promoting monocyte differentiation into macrophages. The resulting macrophages subsequently upregulate MHC class II and promote T-cell polarization toward a T17 phenotype. T17 cells secrete IL-17A, which feeds back to recruit neutrophils, thereby establishing a self-amplifying protumor circuit [73]. Consistently, Jin et al. demonstrated that pulmonary commensal bacteria (e.g., *Staphylococcus aureus* and *Streptococcus* spp.) stimulate macrophages and neutrophils via a MyD88-dependent TLR signaling pathway to produce pro-inflammatory cytokines IL-1β and IL-23, which further promote T-cell proliferation and differentiation into IL-17-producing cells [47].

Beyond these effects, pulmonary microbiota (e.g., *Haemophilus influenzae*) enhance the formation of neutrophil extracellular traps (NETs) [74,75]. NET is negatively associated with CD8^+^ T-cell infiltration and positively correlated with IL-8 [76,77]. NETs have been shown in multiple diseases to physically shield tumor cells from immune cells [77,78] and to impair T-cell motility [78]. In addition, PD-L1 expressed on NETs induces T-cell exhaustion [79]. DNA on NETs may further promote CD8^+^ T-cell dysfunction through activation of the cGAS–STING pathway [80]. Moreover, NETs may activate naïve CD4^+^ T cells via TLR4 signaling, upregulating pro-Treg genes (e.g., *Tgfb1*, *Id3*), downregulating pro-effector T-cell genes (e.g., *Stat4*, *Il6st*), enhancing oxidative phosphorylation, and ultimately promoting CD4^+^ T-cell differentiation into Tregs [81]. NETs have also been reported to correlate positively with CAFs [82]. Collectively, these processes contribute to resistance to ICIs therapy [83,84,85].

With respect to NK cells, the Fap2 protein on the surface of *Fusobacterium nucleatum* can bind the inhibitory receptor TIGIT on NK cells, thereby delivering suppressive signals [86]. Pulmonary commensal bacteria—particularly taxa enriched in tumor tissue—may chronically stimulate NK cells in TME, resulting in increased TIGIT expression on NK cells and upregulated expression of the ligand CD155 on tumor cells. TIGIT–CD155 engagement triggers a cascade characterized by NK-cell functional exhaustion and reduced secretion of cytokines such as IL-2 and IFN-γ, further weakening antitumor immune responses [45]. By contrast, aerosolized *L. rhamnosus* increases CD69 expression on NK cells and upregulates NKG2D, thereby directly enhancing NK-cell activation [72].

In lung adenocarcinoma tissues, *Aspergillus sydowii* has been reported to be significantly enriched; high *A. sydowii* abundance is associated with immunosuppression (e.g., accumulation of PD-1^+^ CD8^+^ T cells) and unfavorable prognosis. β-Glucan, a fungal cell wall component of *A. sydowii*, is recognized by the macrophage surface receptor Dectin-1, activating downstream CARD9 signaling. This pathway induces IL-1β secretion and promotes differentiation and activation of MDSCs. MDSCs suppress cytotoxic T-cell activity through secretion of arginase-1 (ARG1), reactive oxygen species (ROS), and nitric oxide (NO), and further induce expansion of Tregs and accumulation of PD-1^+^ CD8^+^ T cells. Tregs together with PD-1^+^ CD8^+^ T cells establish an immune-tolerant microenvironment, facilitating tumor immune escape [40]. In addition, Streptococcus salivarius and Streptococcus agalactiae activate monocytes to secrete IL-6, IL-12, and TNF-α, which in turn drive Th1/Th17 differentiation [87].

#### 5.1.3. Regulation of Adaptive Immune Cells by Intratumoral Microbiota

Intratumoral microbes have been shown to suppress T-cell function. For instance, the Fap2 protein on *F. nucleatum* binds TIGIT on T cells and transmit inhibitory signals [86]. Conversely, certain microbes enhance T-cell activity: aerosolized *L. rhamnosus* increases CD69 expression on T cells, promoting T-cell activation [72]. Moreover, pulmonary microbiota-mediated upregulation of CXCL9 in cancer and myeloid cells recruits CD8^+^ T cells into the tumor core and enhances the efficacy of nivolumab [66]. The abundance of Streptococcus correlates positively with CD8^+^ T cells [71]. In addition, an intestinally derived *Akkermansia muciniphila* recruits Th1 cells into the TIME and enhance ICI efficacy, potentially via migration from the gut to lung tumors through the circulation [36,59].

Several intratumoral microbes (e.g., *Desulfococcus*, *Terrabacter*, *Bacteroides*, *Proteus*, *Neisseria*, and *Veillonella parvula*) induce expansion of γδT17 cells and promote IL-17 secretion [48,64,73]. IL-17 drives higher expression of inhibitory receptors such as PD-1 and TIM-3 on CD8^+^ T cells, and also reduces PD-L1 expression on tumor cells; together, these changes further suppress T-cell activity and mediate ICI resistance [88]. IL-17 additionally fuels inflammatory remodeling of the TME: IL-17 induces G-CSF expression, promoting neutrophil recruitment into TME [47]. IL-17 directly induces IL-6, G-CSF, and CXCL1 production, attracting tumor-associated neutrophils and rendering them the dominant immune cell population within the tumor [88]. Neutrophils further releases IL-1β, establishing an amplification loop of “myeloid cells → IL-1β → γδ T cells → IL-17” [47,73]. T17 cells also directly promote tumor cell proliferation by secreting IL-22 and amphiregulin [47].

By contrast, other studies have suggested that, in mice with an intact commensal microbiota, commensals sustain γδT17 activity through multiple mechanisms: (i) cytokine regulation, whereby commensals stimulate pulmonary production of IL-6 and IL-23 (potentially from DCs), thereby promoting γδT17 differentiation and IL-17A secretion; and (ii) pattern-recognition pathways, whereby γδ T cells may directly sense bacterial components via receptors such as TLR2/TLR1/Dectin-1, triggering IL-17 production. Pulmonary γδT17 cells may then activate antitumor immune responses (potentially via NK cells and CD8^+^ cells) through IL-17 secretion, thereby suppressing the formation of lung cancer metastatic lesions [61].

#### 5.1.4. Effects of Intratumoral Microbiota on Stromal Cells

**Figure 2:**
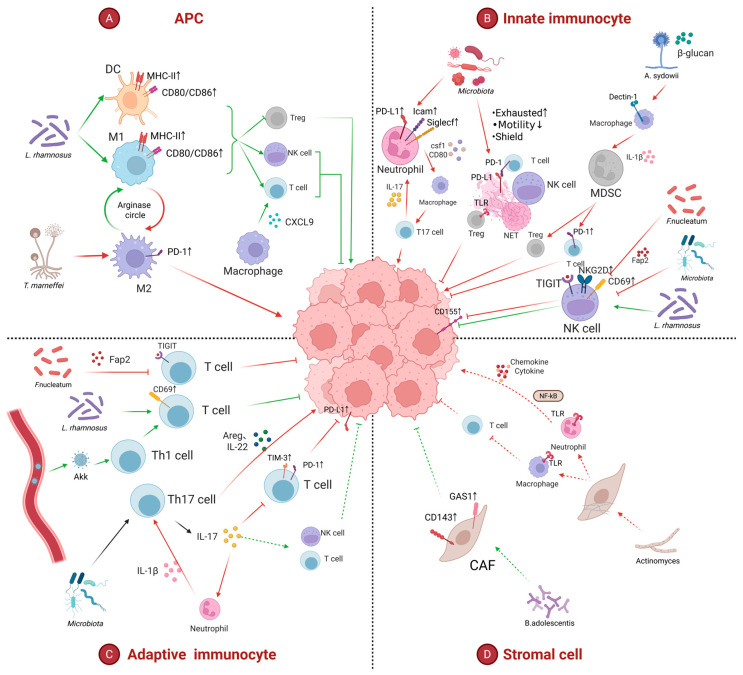
Intratumoral microbiota directly regulate the TIME. The figure illustrates the bidirectional modulation of immune and stromal cells by specific microbial taxa within the TIME. Green arrows indicate antitumor pathways, while red arrows indicate protumor pathways. Solid arrows represent regulatory mechanisms explicitly verified in lung cancer, whereas dashed arrows indicate pathways extrapolated from other tumor types. Arrows indicate changes in expression levels (↑: upregulation; ↓: downregulation). (**A**) APC: *Lactobacillus rhamnosus* enhances antigen presentation by upregulating MHC-II and costimulatory molecules (CD80/CD86) on DCs and M1 macrophages [72]. Conversely, *Talaromyces marneffei* promotes immunosuppressive M2 macrophage polarization and surface PD-1 expression [38]. (**B**) Innate Immunocyte: Fusobacterium nucleatum and other commensals inhibit NK cell cytotoxicity via the Fap2-TIGIT and CD155-TIGIT interaction [45,86]. *L. rhamnosus* activates NK cell by upregulating CD69 and NKG2D [72]. *Aspergillus sydowii* activates MDSCs through the β-glucan/Dectin-1 axis, suppressing T-cell function and activating Tregs [40]. Microbiota-induced neutrophils and NETs facilitate tumor progression by IL-17 secreting, shielding tumor cells, expressing PD-L1, and reducing T-cell motility [48,73,77,78,79,81]. (**C**) Adaptive Immunocyte: *L. rhamnosus* and *Akkermansia muciniphila* (Akk) promote Th1 cell recruitment and T-cell activation [36,59,72]. In contrast, *F. nucleatum* suppresses T cells via TIGIT [86]. Microbial stimulation of the Th17 cell regulates TIME in a bidirectional manner. On one hand, Th17 cells promote tumor cells via promoting their proliferation by secreting Areg, IL-22, and recruiting neutrophil and upregulating TIM-3, PD-1 on T cells via secreting IL-17 [47,88]. On the other hand, IL-17 may activate NK cells and T cells to attack tumor cells [61]. (**D**) Stromal Cell: Actinomyces triggers proinflammatory signaling in CAFs via TLR/NF-κB pathways, whereas *Bifidobacterium adolescentis* induces the expression of the tumor-suppressor GAS1 in CD143^+^ CAFs to restrain tumor growth [90,91]. Created in BioRender. Xue Yang (2025). https://BioRender.com/b8l1ckf.

Microbial diversity has been reported to correlate positively with infiltration of CAFs [18]. Crosstalk between CAFs and immune cells promotes immunosuppression and ultimately contribute to treatment failure [89]. Although the mechanisms in lung cancer remain to be fully defined, bidirectional regulation of CAFs by bacteria has been documented in colorectal cancer. Specifically, actinomycetes locate within CAFs in colorectal tumors, and are recognized by TLR2 in neutrophils and macrophages, leading to activation of downstream NF-κB signaling and modulation of inflammation. Simultaneously, actinomycetes suppress immune responses by inhibiting CD8^+^ T-lymphocyte infiltration [90]. In addition, in colorectal tumors, *Bifidobacterium adolescentis* activates the Wnt/β-catenin pathway and induce high expression of GAS1 in CD143^+^ CAFs; given the tumor-suppressive role of GAS1, this axis may provide novel therapeutic targets for probiotic-based modulation of the TME [91]. Figure 2 provides a schematic overview of effects of intratumoral microbiota on TIME.

### 5.2. Intratumoral Microbial Metabolites as Mediators of Diverse Immunotherapeutic Out-Comes

Metabolites derived from lower respiratory tract microbiota serve as key regulators in reshaping the pulmonary microecosystem in lung cancer [65]. Certain intratumoral microbiota, such as *Bacillus* spp., produce lipid and amino acid metabolites that recruit effective T cells and M1 macrophages, thereby altering TIME [65].

#### 5.2.1. Tryptophan Metabolites

The respiratory microbiota modulate the profile of tryptophan (Trp) metabolites in the lower airways [51]. For example, in lung cancer tissues, N-formylkynurenine is negatively correlated with *Stutzerimonas* and *Altericroceibacterium*, whereas 2-oxoadipate and nicotinic acid are positively correlated with *Chryseobacterium* and *Mycobacteroides*, respectively [51]. Different Trp metabolites, however, appear to exert divergent effects on responses to ICIs. In ICI responders, the relative abundance of serum 3-hydroxyanthranilic acid and multiple Trp derivatives in BALF is increased [51,92], whereas an elevated serum kynurenine (Kyn)/Trp ratio is associated with inferior ICI efficacy [93].

Trp is primarily metabolized via the kyn pathway, the serotonin pathway, and the indole pathway [94]. Indoleamine 2,3-dioxygenase 1/2 (IDO1/IDO2) and tryptophan 2,3-dioxygenase (TDO) are rate-limiting enzymes of the kynurenine pathway and convert Trp into Kyn [94]. The aryl hydrocarbon receptor (AhR) is a central hub linking Trp metabolites to host immune responses. As a ligand-activated transcription factor, AhR can be activated by Kyn, indole, and their derivatives [94]. Distinct ligands trigger “ligand-specific” AhR transcriptional programs, leading to markedly different or even opposite immunological outcomes [95,96].

In certain tumor types, a microbiota–Trp–AhR axis potentiates antitumor immunity. In melanoma, gut-derived *Lactobacillus reuteri* migrates to the tumor and catabolizes dietary Trp into indole-3-aldehyde (I3A), which activates the AhR–cAMP CREB–Blimp-1 signaling cascade in CD8^+^ T cells. This upregulates effector genes such as *Ifng*, *Prf1*, *Gzmb*, and *Tbx21*, as well as inhibitory receptors including *Pdcd1* (PD-1), *Tigit*, and *Lag3*, thereby generating a “high-effector/pre-exhausted” Tc1-like CD8^+^ T-cell subset that enhances ICI efficacy [97]. Despite limited direct evidence in lung cancer, Trp metabolites are generally increased in the lower airways of ICI responders [51,92], suggesting that a similar “microbiota–Trp metabolite–AhR–CD8^+^ T-cell” activation axis may exist in lung tumors.

Conversely, substantial evidence indicates that microbiota-derived Trp metabolites also drive an immunosuppressive TIME and ICI resistance via AhR. IDO1/IDO2/TDO-mediated Trp catabolism generates Kyn, which engages AhR in DCs and skews them toward a tolerogenic phenotype, promoting Treg differentiation and suppressing CD8^+^ T-cell effector function [94,98,99]. In T cells, activation of the Kyn–AhR axis upregulates FoxP3 and represses RORγt, thereby favoring Treg and Th17 generation [100]. In the lung, several nontuberculous mycobacteria and *Pseudomonas aeruginosa* can convert indole back to Trp and subsequently to kynurenine-pathway metabolites such as N-formylkynurenine, contributing to local immunosuppression [101]. Lung-resident *L. reuteri* and *L. murinus* can transform Trp into indole-3-lactic acid (ILA) and I3A, which activate AhR in macrophages and induce *IL-10*, *Arg1*, *IDO1*, and *PD-L1* expression, driving M2-like polarization, decreasing CD8^+^ T cells, and expanding Tregs and MDSCs, thereby establishing a strongly suppressive TIME [98,102]. *Peptostreptococcus*, a genus detected within lung cancer tissues [103], secretes indole-3-acrylic acid (IAA) in endometrial cancer, triggering IL-10 production by macrophages. This, in turn, upregulates IDO1 in tumor cells via the IFN-γ–NF-κB–STAT1–IRF1 axis and amplifies a Trp–Kyn–Treg/M2 positive feedback loop [104]. Small-molecule AhR inhibitors can partially reverse these effects, enhance CD8^+^ T-cell and NK cell activity, and synergize with PD-1/PD-L1 blockade [98,99]. In head and neck squamous cell carcinoma, *Fusobacterium* releases extracellular vesicles carrying tryptophanase into TAMs, where they generate indole and activate the TDO2/AhR pathway. This promotes accumulation of kynurenine-pathway products, AhR nuclear translocation, and transcriptional upregulation of multiple immune checkpoint molecules (PD-1, PD-L1, CTLA-4, LAG-3, etc.), resulting in a highly immune-refractory phenotype [105]. These data collectively indicate that intratumoral microbes can directly reprogram host Trp metabolism to drive TIME remodeling.

Direct evidence for a “tumor-resident microbiota–Trp derivatives–epigenetic regulation–TIME” axis in lung cancer is still lacking; however, studies on intestinal microbiota provide important clues. For instance, *Lactobacillus plantarum*-derived ILA increases H3K27ac at enhancer regions in DCs and reduces Saa3-associated H3K27me3 in CD8^+^ T cells, thereby alleviating T-cell exhaustion [106]. Indole-3-propionic acid (IPA), produced cooperatively by *Lactobacillus johnsonii* and *Clostridium sporogenes*, enhances H3K27 acetylation at Tcf7 super-enhancers, promoting T-cell activation and improving responses to ICI [107]. These findings suggest that Trp and its derivatives produced by lung-resident microbes may similarly remodel the lung cancer TIME via epigenetic mechanisms, representing a promising direction for future investigation.

Overall, although mechanistic evidence in lung cancer for a “tumor-resident microbiota–Trp derivatives–TIME” axis at the levels of cell signaling, epigenetic regulation, and transcriptional reprogramming remains limited, Trp metabolites produced by the lower airway microbiota have strong potential as predictive biomarkers of ICI efficacy and as targets for combination therapy. There is an urgent need to integrate multi-omics and spatial multi-omics analyses in lung cancer cohorts to dissect the precise contributions of distinct Trp metabolic pathways and their specific microbial sources, thereby providing a theoretical basis for the rational remodeling of the TIME and the overcoming of immunotherapy resistance.

#### 5.2.2. Short-Chain Fatty Acids (SCFAs)

In lung cancer, intratumoral microbiota produce a range of short-chain fatty acids (SCFAs), including acetate, propionate, butyrate, and valerate. Compared with adjacent normal lung tissue, SCFA levels are modestly increased in tumor-bearing segments and show significant associations with specific bacterial taxa, displaying a positive correlation with the abundance of Brachyspira hydrosenteriae and a negative correlation with *Pseudomonas* [108]. SCFAs derived from the lung cancer microbiota act on multiple cell types within the TIME, shaping antitumor immunity or immunosuppression through interconnected signaling, epigenetic, and transcriptional mechanisms.

SCFAs are prototypical small-molecule epigenetic regulators. One of their key features is inhibition of HDACs, through which they broadly modulate chromatin accessibility and transcriptional programs [20,109]. SCFAs can inhibit HDAC1/2, leading to increased histone acetylation, a transition of chromatin from a condensed to a more open state, and facilitated access of transcriptional machinery [109]. In T cells, such open chromatin favors transcriptional activation at promoters of effector cytokines (e.g., IFN-γ, TNF-α) and lineage-defining transcription factors (e.g., T-bet, Eomes), thereby epigenetically “locking in” CD8^+^ T-cell effector fate and enhancing antitumor responses [109]. In parallel, SCFAs potentiate phosphorylation of mTOR and its downstream target S6 ribosomal protein, substantially increasing glycolytic flux and driving metabolic reprogramming from a quiescent/memory state toward a high-effector state in T cells [109]. In lung cancer cells, butyrate produced by *Roseburia* suppresses HDAC2 expression and activity, thereby reducing its deacetylation of histone H3K27 in the vicinity of the H19 promoter and leading to transcriptional activation of H19 [20]. Upregulated H19 can inhibit antitumor immunity through multiple pathways and contribute to resistance to immunotherapy [21]. In lung cancer, H19 represses miR-200a, resulting in increased expression of ZEB1 and ZEB2. ZEB1, in turn, induces CD70 upregulation, which drives T-cell exhaustion and is associated with reduced neutrophil counts [110]. ZEB1 can also relieve miR-148a–mediated repression of Rab-dependent exocytosis, promoting the secretion of cytokines that further mediate CD8^+^ T-cell exhaustion [111]. Thus, the axis from *Roseburia*-derived butyrate to HDAC2 inhibition and activation of the H19–ZEB1/2–CD70/Rab pathway constitutes a continuous “metabolite → epigenetics → transcription factor → immune exhaustion” cascade. In macrophages, butyrate enhances the expression of M2 markers such as Arg1 and Ym1, thereby transcriptionally reprogramming macrophages from a proinflammatory M1 phenotype toward an immunosuppressive M2 phenotype, a process that also involves HDAC2 inhibition and downstream gene regulation [20]. These M2-like TAMs secrete IL-10, TGF-β, and related mediators, which suppress effector T-cell function and promote Treg accumulation, effectively translating SCFA-driven gene-expression changes into alterations in cellular composition and function within the TIME [20].

Propionate directly modulates tumor cell survival and death signaling. Propionate induces apoptosis in lung cancer cells by downregulating the survival protein Survivin and upregulating the cyclin-dependent kinase inhibitor p21 [112].

Data on acetate in lung cancer remain limited; however, studies in breast cancer models indicate that acetate can enhance T-cell effector functions [113], suggesting that acetate may exert positive immunoregulatory effects within the TIME by boosting T-cell metabolism and cytotoxic programs [113]. Together with observations that acetate released by *Paenibacillus odorifer* may act as a protective, anti-lung-cancer factor [114], these findings highlight the need to further dissect acetate-associated gene-expression signatures and immune effects in lung cancer, in order to clarify its role within the intratumoral SCFA network.

#### 5.2.3. Lactate-Mediated Immunosuppression

Intratumoral microbiota in lung cancer, including anaerobic and certain commensal species, produce large amounts of lactate. The resulting acidic TME, in turn, favors colonization of anaerobic microbes, which further sustain local acidification through lactate production, thereby establishing a self-reinforcing “acidic niche–anaerobic microbiota–lactate” loop [115]. Multi-omics analyses linked increased lactate metabolism within the TIME to immunosuppressive features, including augmented infiltration of M2 macrophages and Tregs, elevated indices of immune evasion such as TIDE scores, and reduced response rates to ICIs [116,117].

Through multiple signaling pathways, lactate produced by intratumoral microbes such as *Lactobacillus iners*, *Staphylococcus* spp., and *Escherichia coli* modulates the TIME. *L. iners* generate L-lactate within the TME. Lactate regulate intracellular NF-κB, SIRT3/ROS/HIF-1α, FGFR, ErbB3/HER2/3 and p53/p73-dependent apoptotic pathways, thereby promoting tumor-cell proliferation and survival [19]. These signaling alterations not only raise the threshold for cell death but also create a context for immune evasion. In lung cancer, intratumoral *Staphylococcus* secrete lactate, which upregulates the monocarboxylate transporter MCT1 on tumor cells, enhances lactate uptake, inhibits PHD2 activity, and stabilizes hypoxia-inducible factor 1α, leading to activation of downstream hypoxia pathways such as LDHA, ENO1, and VEGFA [118]. Furthermore, lactate taken up by CAFs, induces nuclear translocation of Nusap1, and facilitates recruitment of the JUNB–FRA1–FRA2 transcriptional complex to the DESMIN promoter, resulting in DESMIN upregulation. Activated DESMIN^+^ CAFs secrete IL-8, which recruits and polarizes M2 TAMs. M2 TAMs in turn, further reinforce the immunosuppressive milieu through secreting IL-10, TGF-β, and Arg1 [22]. In addition, lactate activates the GPR81 receptor on lung cancer cells, reduces cAMP/PKA activity, attenuates Hippo-pathway–mediated inhibition of TAZ, and promotes TAZ nuclear translocation and interaction with TEAD transcription factors, thereby upregulating PD-L1 expression, impairing CD8^+^ T-cell function, and facilitating immune escape [119].

Lactylation exerts epigenetic effects on both histone and non-histone proteins and contributes to suppressive TIME. Beyond its role as a metabolic by-product, lactate serves as an acyl-group donor for protein lactylation. Lactylation of APOC2 at lysine 70 markedly increases its stability and promotes the release of free fatty acids (FFAs) [120]. As a major energy source for immune cells, particularly Tregs, FFAs support Treg accumulation and contribute to ICI resistance [120]. Thus, APOC2-K70 lactylation provides a mechanistic link between lipid metabolism and immunometabolic reprogramming. Lactate also drives histone lactylation, altering chromatin architecture and broadly increasing chromatin accessibility [22]. In lung cancer, H3K18la is enriched at the promoter of the nucleoporin POM121 and enhances its transcription [121]. As a key component of the nuclear pore complex, POM121 accelerates nuclear import of MYC, leading to its accumulation in the nucleus [121]. Nuclear MYC then directly binds to the CD274 (PD-L1) promoter, driving PD-L1 transcription and expression [121]. This “lactate–H3K18la–POM121–MYC–PD-L1” cascade represents an example of lactate amplifying immune-checkpoint signaling through epigenetic mechanisms. Moreover, *E. coli*–derived lactate can lactylate the pattern-recognition receptor RIG-I in macrophages, thereby inhibit RIG-I-MAVS-NF-ĸB signaling, impairing NF-κB recruitment to the Nlrp3 promoter and promoting M2 polarization. These lactylated macrophages secrete TGF-β and IL-10, upregulate PD-1 expression on Tregs, and suppress CD8^+^ T-cell proliferation and function, collectively generating a highly immunosuppressive microenvironment [23].

At the genetic level, lactate coordinately reshapes multiple metabolic pathways within the TIME and in tumor cells. It upregulates genes involved in glycolysis, glutamate metabolism, and galactose metabolism, thereby reinforcing the Warburg effect [19]. In parallel, it downregulates gene programs associated with DNA damage responses, initiation of DNA replication, G2/M and S-phase checkpoints, and E2F targets [19]. This combination of “metabolic enhancement with attenuated DNA surveillance” provides tumor cells with a survival advantage under stress and fosters therapeutic resistance.

Microorganisms also indirectly shape the TIME by modulating the expression of lactate-metabolism–related genes in lung cancer tissue. The abundance of *Lachnoclostridium* has been found to correlate strongly with the expression profiles of a panel of key lactate-metabolism genes [116]. Mediation analyses suggest that *Lachnoclostridium* may suppress CD8^+^ T cells by upregulating TUFM, GFM1, CHEK2, NDUFA10, and AGK; suppress CD4^+^ T cells via upregulation of TUFM, PNPLA2, GFM1 and CHEK2; attenuate NK-cell activity through GFM1; and may be associated with the recruitment of M2 macrophages and Tregs [116]. This microbiota–gene–immune-cell axis reveals a form of “remote control” by which intratumoral microbes remodel immune-cell composition and function through a lactate metabolism–centered gene network.

#### 5.2.4. Reactive Oxygen Species (ROS)

ROS are highly reactive molecules generated as by-products of molecular oxygen (O_2_) metabolism during aerobic respiration, mainly including superoxide anion (O_2_^•−^), singlet oxygen (^1^O_2_), hydrogen peroxide (H_2_O_2_), and hydroxyl radicals (•OH) [24,122]. ROS can activate stress-response pathways and oncogenes, downregulate tumor suppressor genes, induce cellular and tissue damage, and thereby promote tumor initiation, promotion, and progression [24]. Their biological effects are concentration-dependent: at low levels, ROS maintain normal cellular functions; at slightly elevated levels, they promote tumorigenesis; whereas at high levels, they exert cytotoxic effects [123]. The sources of ROS are divided into endogenous and exogenous. Endogenous ROS are primarily produced by mitochondria, NADPH oxidases, and peroxisomes, whereas exogenous ROS are triggered by environmental factors such as nitrogen dioxide (NO_2_), sulfur dioxide (SO_2_), carbon monoxide (CO), and particulate matter in air (e.g., cigarette smoke) [124]. Recent studies have further identified microorganisms as important contributors to ROS generation.

Microorganism-derived ROS are produced through two main mechanisms: direct and indirect. On the one hand, microorganisms directly generate ROS. For example, *Enterococcus faecalis* releases superoxide and H_2_O_2_ [125]. The outer membrane protein FomA of *Fusobacterium nucleatum* form a complex with Cu(II), inducing abundant extracellular ROS production [126]. In lung, *Streptococcus pneumoniae*, which enriches in lung cancer, produce high concentrations of H_2_O_2_ in the pulmonary environment, which may contribute to its malignant biological behavior [127,128]. On the other hand, microorganisms indirectly induce ROS production by activating host cells. *Bacteroides fragilis*–derived *B. fragilis* toxin (BFT) upregulates spermine oxidase (SMO) in colonic epithelial cells and thereby induces SMO-dependent ROS generation [24]. BFT also stimulates ROS production in DCs [129]. Invasive HtrA-positive Escherichia coli promotes excessive ROS production in epithelial cells, contributing to cancer initiation [130]. Although direct measurements of bacteria-derived ROS in lung cancer tissues are still lacking, the partial overlap between the lung and gut microbiota, the existence of the gut–lung axis, and the known characteristics of pulmonary pathogens strongly suggest that intratumoral microbiota–derived ROS in lung cancer are highly plausible and warrant further investigation.

**Figure 3:**
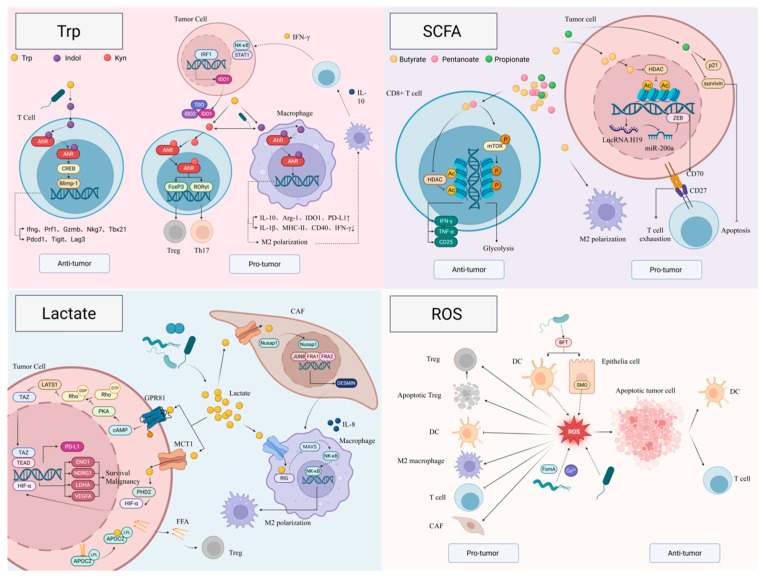
Indirect regulation of the TIME by microbial metabolites. The figure depicts the complex immunomodulatory networks driven by microbiota-derived metabolites. Solid arrows indicate regulatory mechanisms explicitly verified in lung cancer, whereas dashed arrows represent pathways extrapolated from other tumor types. Pointed and blunt-ended arrows indicate promotion and inhibition, respectively. Tryptophan (Trp): The effects of Trp metabolites depend on the ligand-specific activation of the AhR. Indoles promote a “high-effector” CD8^+^ T-cell phenotype via the AhR–CREB–Blimp-1 axis (Anti-tumor). Conversely, in T cells, the Kyn-AhR axis upregulates FoxP3, inhibits RORγt, and promotes the production of Treg and Th17. Indole activates AhR in macrophages, induces M2 polarization, and produces IL-10. Subsequently, it upregulates IDO1 in tumor cells through the IFN-γ-NF-κB-STAT1-IRF1 signaling axis, amplifying the Trp-Kyn-Treg/M2 positive feedback loop (Pro-tumor). Short-Chain Fatty Acids (SCFAs): SCFAs function primarily as HDAC inhibitors. On one hand, they epigenetically enhance effector gene expression and mTOR signaling in CD8^+^ T cells. On the other hand, SCFAs inhibit HDACs in tumor cells to activate the H19–ZEB–CD70 axis, causing T-cell exhaustion. Propionate induces tumor cell apoptosis via p21 upregulation. SCFAs promote M2 polarization. Lactate: Lactate fosters an immunosuppressive niche through signaling and protein lactylation. It activates GPR81 on tumor cells to upregulate PD-L1 via TAZ/TEAD. Lactate stabilizes HIF1α protein and activates downstream hypoxia pathways. Lactylation of APOC2 in tumor cells (releasing FFAs) promotes Tregs. M2 polarization and Treg accumulation. Lactate also stimulates CAFs to recruit M2 macrophages via IL-8. Lactate upregulates DESMIN expression in CAF, which secretes IL-8, recruiting and polarizing M2-type TAMs. ROS: ROS exerts dose-dependent, dual effects. Microbial ROS (induced by toxins like BFT or FomA) generally promotes resistance by impairing DC maturation, inducing T-cell apoptosis, and supporting M2 macrophage, CAF and Treg apoptosis and Tregs (Pro-tumor). However, high-level ROS can trigger immunogenic tumor cell death and activate DCs to prime T-cell responses (Anti-tumor). Created in BioRender. Xue Yang (2025). https://BioRender.com/ij1v057.

Intratumoral ROS profoundly remodel TIME and exert bidirectional effects on antitumor immunity and immunotherapy. On the one hand, ROS in TIME promote resistance to immunotherapy. Tregs in the TIME are highly sensitive to ROS; elevated ROS levels in TME trigger Treg apoptosis. Notably, apoptotic Treg cells exhibit even stronger immunosuppressive effects. Therefore ROS-induced Treg apoptosis in turn enhances their suppressive effects and contributes to resistance to PD-L1 inhibitors [131]. BFT from *B. fragilis* drives ROS generation in DCs, activate the ERK/p38 MAPK–Nrf2–HO-1 axis, impair DC maturation, and promote immune tolerance [129]. In lung adenocarcinoma, increased ROS levels have been reported to correlate negatively with cytotoxic T-cell infiltration, DC maturation, and antigen-presenting capacity, and to predict resistance to PD-1/PD-L1 inhibitors [132]. Excessive ROS induce apoptosis of tumor-infiltrating T cells and hinder the formation and maintenance of memory CD8^+^ T cells [133]. In addition, ROS promote Treg differentiation, maintain MDSCs in an immature state, drive M2 polarization of macrophages, and facilitate the differentiation of CAFs into myofibroblast-like phenotypes. These changes collectively suppress T-cell proliferation, enhance immune checkpoint expression, and increase the production of immunosuppressive cytokines, thereby fostering an immunosuppressive TIME [133]. On the other hand, ROS within the TIME can induce oxidative stress, leading to protein, lipid, and DNA damage, cell-cycle arrest, and apoptosis, thereby potentially suppressing tumorigenesis or killing tumor cells [122] and reshaping the TIME to favor antitumor immunity. Moderate levels of ROS participate in multiple steps of antitumor immune responses, including DC antigen uptake and presentation, as well as T-cell activation and proliferation [133]. Excessive ROS can also be therapeutically exploited in lung cancer. Sun et al. [134] demonstrated that high-dose ascorbic acid in lung cancers with primary resistance to immunotherapy and LKB1 deficiency can markedly increase intracellular ROS levels, thereby inducing pyroptosis of lung cancer cells, promoting DC maturation, and enhancing recruitment and proliferation of T cells. These changes collectively reprogram the TIME and reverse immunotherapy resistance. Figure 3 provides a schematic overview of the mechanisms of microbial metabolites’ regulation on TIME.

### 5.3. Bacterial Extracellular Vesicles (BEVs)

BEVs are nanoscale vesicles (20–400 nm) secreted by Gram-positive and Gram-negative bacteria, containing proteins, lipids, polysaccharides, nucleic acids, and metabolites [135]. BEVs have dual effects. On one hand, BEVs (such as those from *Bacillus* licheniformis) inhibit cell viability and proliferation by increasing ROS and reducing glutathione, directly inducing death in lung cancer cells [136]. On the other hand, BEVs also facilitate immune escape by delivering PD-L1, skewing macrophages toward M2 polarization, suppressing NK cytotoxicity via delivering TGF-β, and inhibiting CD8^+^ T-cell activation through miR-23a cargo [135].

Conversely, beneficial effects have been observed. After entering circulation, gut commensal *Bifidobacterium*-derived extracellular vesicles (*Bif.*BEVs) containing lipoteichoic acid are up-taken by lung cancer cells predominantly via dynamin-dependent endocytosis and upregulate PD-L1 expression through TLR4-NF-κB pathway. Those *Bif.*BEVs can also up-regulate IL-2 and IFN-γ, and recruit CD8^+^/CD3^+^ T-cell while downregulating Ras–MAPK and TGF-β pathways, thereby improving anti-PD-1 efficacy [137].

### 5.4. Antimicrobial Peptides (AMPs)

AMPs are widely distributed molecules in diverse organisms that can kill a broad spectrum of bacteria, fungi, and viruses. They function as both microbicidal agents and immunomodulators within the innate immune system and interact closely with the adaptive immune response; more recently, they have also been implicated in cancer biology [138]. The sources of AMPs include bacteriophages, bacteria, fungi, plants, animals, and humans [139]. Among these, AMPs that exhibit selective cytotoxicity toward cancer cells are termed anticancer peptides.

In the context of malignancy, AMPs directly kill cancer cells, activate immune effector cells, and modulate the TIME [140]. However, studies specifically addressing the roles of microbially secreted AMPs within the TIME of lung cancer are currently lacking. Consequently, the following discussion is largely extrapolated from data obtained in other tumor types.

Overall, microbial AMPs possess both direct antitumor cytotoxic activity and immunomodulatory capacity. On the one hand, AMPs directly kill tumor cells by inducing apoptosis, causing cell-cycle arrest, inhibiting cell migration, and disrupting cellular membrane integrity [141]. *Escherichia coli*, identified as intratumoral microbiota in lung cancer and correlating with improved responses to ICIs [60], can secrete colicin N to induce apoptosis in multiple tumor types, including lung cancer [142]. Nisin, produced by lactic acid bacteria, displays selective cytotoxicity against lung cancer cells and can inhibit tumor cell migration and proliferation [143,144]. Other microbial AMPs, including enterocin, laterosporulin 10, colicins, microcin E492, pediocin, pyocin, and bovicin, also exhibit cytotoxic and antiproliferative activity against a variety of cancer cell types [144].

On the other hand, AMPs exert immunomodulatory effects, both pro-inflammatory and anti-inflammatory. First, AMP-induced tumor cell apoptosis releases DAMPs and tumor antigens, which promotes DCs antigen presentation, thereby promoting the priming of CD8^+^ T cells. Second, AMPs directly acts on immune cells within the TIME. Aureocin A53, produced by *Staphylococcus*, activates murine macrophage cell lines to produce TNF and, in synergy with IFN-γ, induces NO production, thereby amplifying inflammatory responses [145]. Acidocin A, secreted by *Lactobacillus acidophilus,* induces the production of multiple inflammatory mediators (IL-6, TNF-α, MIG/CXCL9, MCP-1/C-C motif CCL2, MCP-3/CCL7, and MIP-1β) in monocytes, while suppressing certain anti-inflammatory factors such as IL-1 receptor antagonist and MDC/CCL22, indicating potent pro-inflammatory properties [146]. Conversely, other AMPs exhibit anti-inflammatory effects. Nisin from *Lactococcus lactis* promotes lymphocyte apoptosis and the formation of NETs [147]. Plantaricin-type AMPs can inhibit the release of NO and ROS from macrophages following LPS stimulation, further supporting their anti-inflammatory potential [148].

Taken together, these indirect lines of evidence support the notion that microbe-derived AMPs may reduce tumor burden in vivo through direct cytotoxicity while simultaneously shaping the TME by modulating immune-cell function and remodeling the cytokine milieu. It is therefore plausible that, if intratumoral microbes in lung cancer produce AMPs with similar properties, they could influence TIME and contribute to the efficacy immunotherapies. Future studies integrating intratumoral microbiome derived AMP expression and function is necessary.

## 6. Intratumoral Microbiota and Immune-Related Adverse Events (irAEs)

Immune checkpoint inhibitors often induce immune-related adverse events (irAEs) affecting multiple organs. Among them, checkpoint inhibitor–related pneumonitis (CIP) is the most severe. Interestingly, the occurrence of irAEs has been linked to better therapeutic responses [69].

To date, only CIP has been reported in association with lung cancer microbiota. Increased abundance of Proteobacteria and Firmicutes in the lower airway is correlated with CIP [149]. Yu et al. [150] demonstrated significantly higher microbial α-diversity in BALF samples from CIP patients. Enriched taxa included *Vibrio*, *Halomonas*, *Mangrovibacter*, *Paracoccus*, *Salinivibrio*, *ZOR0006*, *Mitochondria*, and *Sphingobium*. CIP-associated BALF also displayed elevated fatty acid metabolism. Neutrophils were negatively associated with *Lachnospiraceae*_NK4A136_group, *Christensenellaceae*_R_7_group, and *Akkermansia*; CD8^+^ T cells were negatively correlated with *Brevundimonas* but positively correlated with *Clostridia*_UCG_014 and *Parabacteroides*. CD4^+^ T cells were strongly positively correlated with *Weissella*, while B cells correlated positively with *Anaerostipes* and negatively with *Staphylococcus*. In addition, the metabolite lauroylcarnitine was shown to activate T cells, inducing IFN-γ and TNF-α secretion.

Currently studies on intratumoral-microbiota-related irAEs in lung cancer remain scarce, highlighting a critical area for future investigation.

## 7. Clinical Translation and Intervention Strategies

### 7.1. Predictive Value of Microbial Biomarkers

As summarized in Table 1, intratumoral microbiota obtained from multiple sources are predictive of immunotherapy response in lung cancer. Microorganisms derived from BALF have been shown to correlate with ICI outcomes. At the genus level, *Fusobacterium*, *Chryseobacterium*, *Haemophilus influenzae*, and *Neisseria perflava* were associated with poor response [65,68,70], whereas *Bacillus*, *Streptomyces*, and *Veillonella dispar* correlated with favorable ones [51,65,70]. At the phylum level, Firmicutes were enriched in responders, while Bacteroidota and Proteobacteria were enriched in non-responders [65,70]. In anti–PD-1 therapy, *Staphylococcus* can predict better outcomes with an ROC AUC of 0.8209 (*p* < 0.05), while *Nostoc* reached an AUC of 0.7540 (*p* < 0.05) [51]. Saliva-based analyses revealed that, at the phylum level, responders harbored more Bacteroidota and fewer Firmicutes. At the genus level, responders carried more *Fusobacterium* and *Porphyromonas* but fewer *Streptococcus* [69]. The abundance of *Gemella* is the strongest predictor of non-response, whereas *Lachnoanaerobaculum* was the best predictor of positive response [69]. In microbiota isolated from sputum, *Atopobium* and *Streptococcus* are predictive of favorable immunotherapy efficacy, with ROC AUC values of 0.67 (*p* = 0.04) and 0.77 (*p* < 0.01), respectively [71]. Tumor biopsy and surgical specimens indicated that intratumoral Escherichia was significantly associated with longer overall survival following ICI therapy [60], while *Gammaproteobacteria* and *Aspergillus sydowii* are related to poor outcomes [40,67]. Bioinformatics database analyses identified *Fusobacterium* as a marker of poor therapeutic efficacy [18].

### 7.2. Microbiota-Targeted Intervention Strategies

#### 7.2.1. Probiotics Modulation

Probiotics enhance ICI efficacy and improve prognosis [151,152,153]. Le Noci et al. [72] demonstrated that aerosolized *Lactobacillus rhamnosus* promote maturation of resident lung APCs and reduce M2-polarized TAMs, thereby increasing activation of effective cells such as T cells and NK cells, ultimately suppressing pulmonary metastasis. Another study confirmed that both live or inactivated *Lactobacillus rhamnosus* aerosolization is associated with increased infiltration of B cells, NK cells, and CD4^+^ T cells, alongside reduced Tregs and M2 macrophages in lung adenocarcinoma [154]. The probiotic *Clostridium butyricum* significantly prolongs PFS and OS, with efficacy also observed in patients receiving antibiotics [155]. Probiotic *Bifidobacterium* induced apoptosis of NSCLC cells, and increase invasiveness by downregulating MMP-9. Additionally, *Bifidobacterium* activate DCs, promoted T-cell recruitment and function, and enhanced the therapeutic efficacy of PD-L1 inhibitors [156,157].

#### 7.2.2. Rational Use of Antibiotics

Antibiotic exposure during immunotherapy is associated with lower survival [158,159], likely due to its disruption of airway and gut microbiota. The impact of antibiotics on ICI efficacy in advanced NSCLC appears to depend on PD-L1 expression. A negative effect of antibiotic use was observed in patients with PD-L1 ≥ 50%, but not in those with PD-L1 < 50% [160]. Broad-spectrum antibiotics impair γδ T-cell antitumor activity [64], and their use prior to therapy should be carefully evaluated.

Mechanistically, antibiotics induce pulmonary dysbiosis, reduce CXCL9 production in TME, impair T-cell infiltration induced by anti–PD-1 therapy, and ultimately diminish therapeutic efficacy [66]. Antibiotic use is correlated with lower ICI response [69]. Conversely, Wang et al. [45] reported that antibiotics could suppress protumor microbial factors in lung tumor, although prolonged antibiotic exposure may induce intratumoral bacterial resistance therefore exhibiting no accelerated tumor growth. Notably, lung microbiota, altered by aerosolized vancomycin/neomycin, reduce Tregs and enhancing T-cell and NK-cell activation [72]. In addition, antibiotic-induced loss of microbial diversity is linked to increased incidence of irAEs [161].

#### 7.2.3. Engineered Bacteria Enhances Immunotherapy

Several bacteria can be genetically engineered to serve as immunostimulatory agents, reshaping the TIME and enhancing immunotherapy efficacy. A strong positive correlation between *Pasteurella* abundance and CD8^+^ T-cell infiltration in lung cancer suggests its potential as a therapeutic vaccine candidate [62]. Moreover, lung-resident microbes can drive tumor progression through the myeloid cell–IL-1β–γδ T-cell–IL-17 axis, indicating that disrupting this pathway may provide therapeutic benefit [70]. Below, we summarize representative engineered bacterial strategies:

(1) *Listeria monocytogenes*:

ADXS-503, an engineered *Listeria monocytogenes* strain expressing 22 tumor antigens, is shown to induce innate and adaptive immune responses and reverse resistance in patients progressing on prior pembrolizumab, with favorable safety and tolerability [162,163]. Another engineered *Listeria monocytogenes*, JNJ-64041757, which expresses human mesothelin, can induce cellular immunity against mesothelin-expressing tumor. However its efficacy alone or with nivolumab is limited [164].

(2) *Klebsiella pneumoniae*:

QBKPN, derived from inactivated *Klebsiella pneumoniae*, induces M1 macrophage polarization and enhances NK cell–mediated antitumor activity through NKG2D signaling [165].

(3) *Salmonella typhimurium*:

R-GEM/VNP-IFNγ, a macrophage–*Salmonella* typhimurium fusion engineered to express IFN-γ, preferentially accumulates in pulmonary metastases, depleting M2 macrophages, MDSCs, and Tregs, while increasing M1 macrophages, CD8^+^ T cells, and DCs [166]. Another strain, YB1, is oxygen-sensitive (<0.5%) strain, therefore it selectively replicates in hypoxic tumor regions, inducing regression while dead in normal tissues [167]. YB1 activated NK cells to release IFN-γ, promoting NK cell recruitment and inhibiting metastasis [168].

VNP-C-C is engineered VNP20009 strain expressing CCL2 and CXCL9. VNP can induce immunogenic cell death in tumor cells and trigger the activation of the cGAS/STING pathway, initiating downstream IFN-I and proinflammatory cytokine production, which in turn amplifies tumor immunogenicity. CCL2 primarily regulates the activation of Macrophages and DCs, whereas CXCL9 enhances T-cell infiltration and directs effector T cells, thereby amplifying the immune responses induced by ICD. Altogether VNP-C-C exerts improvement in the immunosuppressive TME and exhibits promising antitumor potential [169].

(4) *Bacillus* Calmette–Guérin (BCG):

BCG can stimulate immune responses. In murine models, intravenous BCG activates and recruits CD4^+^ T cells, DCs, CD8^+^ T cells, and NK cells, enhancing the efficacy of PD-L1 blockade [170]. Early clinical trials in the 1970s showed survival benefit in patients treated with BCG–CWS. A macrophage membrane–coated BCG formulation (M@BCG) improves BCG accumulation in lung tumors, promotes TAM uptake of BCG, increases proinflammatory cytokine production, enhances M1 macrophage and CD8^+^ T-cell infiltration, upregulates PD-L1, and improves ICI efficacy with favorable safety [171].

## 8. Limitations and Novelty

This review has several limitations. First, the field of intratumoral microbiota in lung cancer is still emerging, and most available data come from small, single-center or retrospective studies, which may introduce selection and publication bias. Second, there is substantial heterogeneity in sampling procedures, contamination control, sequencing platforms and bioinformatic pipelines, limiting the comparability of microbial profiles across studies and preventing firm conclusions about specific taxa. Third, many mechanistic insights are extrapolated from preclinical models or other tumor types, so causal relationships between intratumoral microbes, TME remodeling and immunotherapy efficacy in lung cancer remain to be validated in large, prospective clinical trials.

Despite these limitations, this review is novel in that it specifically focuses on intratumoral, rather than gut, microbiota in lung cancer and integrates current evidence on how microbial alterations modulate the TME and shape responses to immune checkpoint blockade. By summarizing clinical, experimental and multi-omics findings and highlighting testable hypotheses for microbiota-based biomarkers and interventions, our work provides a conceptual framework to guide future translational studies and personalized therapeutic strategies in lung cancer.

## 9. Conclusions

Intratumoral microbiota in lung cancer are emerging as active regulators of the TIME, capable of both enhancing and impairing responses to immune checkpoint inhibitors. Evidence to date indicates that microbial composition, metabolites, and bacterial extracellular vesicles can reshape immune cell infiltration and function, thereby influencing treatment efficacy and resistance. These findings support the potential of intratumoral microbial features as predictive biomarkers and as therapeutic targets for approaches such as rational antibiotic use, probiotics, and engineered bacterial therapies. However, most current data are heterogeneous and largely correlative, and standardized methods to define causality and clinical utility are still lacking. Future studies integrating spatial multi-omics, mechanistic validation, and prospective clinical trials are essential to refine microbiota-based strategies and ultimately improve immunotherapy outcomes in patients with lung cancer.

## Figures and Tables

**Table 1:** Relationship between intratumoral microbiota and response to immunotherapy in lung cancer.

Phylum	Genus	Study Design	Sample Type	Cohort Size	Lung Cancer Subtype	Line of Treatment/Regimen	Clinical Endpoint	Prediction	Ref.
Fusobacteria	*Fusobacterium*	Prospective study	BALF	80	LUAD, LUSC	1L/Anti-PD-1 monotherapy	OS, PFS	Poor outcomes	[68]
		Retrospective analysis	Tumor biopsies	63 NSCLC subsets in 4160 biopsies	NSCLC	NR/ICI-monotherapy	OS, PFS	Poor outcomes	[18]
		Prospective study	Saliva, sputum	55	Stage III–IV NSCLC (LUAD 60%, LUSC 40%)	First-line and subsequent lines/pembrolizumab, atezolizumab, durvalumab, nivolumab	OS, PFS	Better outcomes	[69]
Firmicutes	*-*	Prospective study	Saliva, sputum	55	Stage III–IV NSCLC (LUAD 60%, LUSC 40%)	First-line and subsequent lines/pembrolizumab, atezolizumab, durvalumab, nivolumab	OS, PFS	Poor outcomes	[69]
		Retrospective study	BALF	26	NSCLC	NR/ICI-monotherapy	Clinical response, PFS, OS	Better outcomes	[65]
		Prospective study	BALF	84	NSCLC (76.2% LUAD: 23.8% LUSC)	NR/immunotherapy (11 in 84)	Clinical response	Better outcomes	[70]
	*Streptococcus*	Prospective study	Sputum and stool	75	Metastatic NSCLC	Subsequent lines/anti-PD-1 monotherapy	Clinical response, PFS	Better outcomes	[71]
		Prospective study	Saliva, sputum	55	Stage III–IV NSCLC (LUAD 60%, LUSC 40%)	First-line and subsequent lines/pembrolizumab, atezolizumab, durvalumab, nivolumab	OS, PFS	Poor outcomes	[69]
	*Gemella*	Prospective study	Saliva, sputum	55	Stage III–IV NSCLC (LUAD 60%, LUSC 40%)	First-line and subsequent lines/pembrolizumab, atezolizumab, durvalumab, nivolumab	OS, PFS	Poor outcomes	[69]
	*Staphylococcus*	Retrospective study	BALF	56	NSCLC	NR/anti-PD-1 immunotherapy	Clinical response, PFS, OS	Better response	[51]
	*Lachnoanaerobaculum*	Prospective study	Saliva, sputum	55	Stage III–IV NSCLC (LUAD 60%, LUSC 40%)	First-line and subsequent lines/pembrolizumab, atezolizumab, durvalumab, nivolumab	OS, PFS	Better outcomes	[69]
	*Veillonella*	Prospective study	BALF	84	NSCLC (76.2% LUAD: 23.8% LUSC)	NR/immunotherapy (11 in 84)	Clinical response	Better outcomes	[70]
	*Bacillus*	Retrospective study	BALF	26	NSCLC	NR/ICI-monotherapy	Clinical response, PFS, OS	Better outcomes	[65]
Bacteroidetes	*-*	Prospective study	BALF	84	NSCLC (76.2% LUAD: 23.8% LUSC)	NR/immunotherapy (11 in 84)	Clinical response	Poor outcomes	[70]
		Retrospective study	BALF	26	NSCLC	NR/ICI-monotherapy	Clinical response, PFS, OS	Poor outcomes	[65]
		Prospective study	Saliva, sputum	55	Stage III–IV NSCLC (LUAD 60%, LUSC 40%)	First-line and subsequent lines/pembrolizumab, atezolizumab, durvalumab, nivolumab	OS, PFS	Poor outcomes	[69]
	*Chryseobacterium*	Retrospective study	BALF	26	NSCLC	NR/ICI-monotherapy	Clinical response, PFS, OS	Poor outcomes	[65]
	*Porphyromonas*	Prospective study	Saliva, sputum	55	Stage III–IV NSCLC (LUAD 60%, LUSC 40%)	First-line and subsequent lines/pembrolizumab, atezolizumab, durvalumab, nivolumab	OS, PFS	Better outcomes	[69]
	*Sediminibacterium*	Retrospective study	BALF	26	NSCLC	NR/ICI-monotherapy	Clinical response, PFS, OS	Poor outcomes	[65]
Proteobacteria	*-*	Prospective study	BALF	84	NSCLC (76.2% LUAD: 23.8% LUSC)	NR/immunotherapy (11 in 84)	Clinical response	Poor outcomes	[70]
		Prospective study	BALF	12	NSCLC	Second- or later-line treatment/Nivolumab (anti-PD-1 antibody)	Clinical response, PFS	Poor outcomes	[66]
	*Neisseria*	Prospective study	BALF	84	NSCLC (76.2% LUAD: 23.8% LUSC)	NR/immunotherapy (11 in 84)	Clinical response	Poor outcomes	[70]
	*Haemophilus*	Prospective study	BALF	84	NSCLC (76.2% LUAD: 23.8% LUSC)	NR/immunotherapy (11 in 84)	Clinical response	Poor outcomes	[70]
	*Escherichia*	Retrospective analysis	Tumor tissue biopsies	Discovery cohort: 958; Validation cohort: 772	NSCLC	NR/ICI therapy	OS, PFS	Better outcomes	[60]
	*Sphingomonas*	Retrospective study	BALF	26	NSCLC	NR/ICI-monotherapy	Clinical response, PFS, OS	Poor outcomes	[65]
Actinobacteria	*Strepto-myces*	Retrospective study	BALF	56	NSCLC	NR/anti-PD-1 immunotherapy	Clinical response, PFS, OS	Better outcomes	[51]

**Abbreviations:** BALF, bronchoalveolar lavage fluid; LUAD, lung adenocarcinoma; LUSC, lung squamous cell carcinoma; NSCLC, non-small cell lung cancer; 1L, first-line; ICI, immune checkpoint inhibitor; anti-PD-1, anti-programmed cell death protein 1; PFS, progression-free survival; OS, overall survival; NR, not reported.

## Data Availability

No new data were created or analyzed in this study. Data sharing is not applicable to this article.

## Abbreviations

The following abbreviations are used in this manuscript:

AhR	Aryl hydrocarbon receptor
AMPs	Antimicrobial peptides
APCs	Antigen-presenting cells
ARG-1	Arginase-1
BALF	Bronchoalveolar lavage fluid
BCG	*Bacillus* Calmette–Guérin
BEVs	Bacterial extracellular vesicles
BFT	*B. fragilis* toxin
Bif.BEVs	Bifidobacterium-derived extracellular vesicles
CAFs	Cancer-associated fibroblasts
CIP	Checkpoint inhibitor–related pneumonitis
CTLA-4	Cytotoxic T-lymphocyte–associated protein 4
DCs	Dendritic cells
EBV	Epstein–Barr virus
FFAs	Free fatty acids
FDA	Food and Drug Administration
ICI	Immune checkpoint inhibitor
HPV	human papillomavirus
IDO	Indoleamine-2,3-Dioxygenase
irAEs	Immune-related adverse events
IPA	indole-3-propionic acid
ILA	indole-3-lactic acid
I3A	indole-3-aldehyde
IAA	indole-3-acrylic acid
Kyn	Kynurenine
LPS	Lipopolysaccharides
LUAD	lung adenocarcinoma
LUSC	lung squamous cell carcinoma
MDSCs	Myeloid-derived suppressor cells
NETs	neutrophil extracellular traps
NK cells	Natural killer cells
NSCLC	Non-small cell lung cancer
OS	Overall survival
PD-1	Programmed cell death protein 1
PD-L1	Programmed death-ligand 1
PFS	Progression-free survival
SCFAs	Short-chain fatty acids
SCLC	Small cell lung cancer
SMO	Spermine oxidase
ROS	reactive oxygen species
TAMs	Tumor-associated macrophages
TAZ	transcriptional coactivator with PDZ-binding motif
TDO	tryptophan 2,3-dioxygenase
TIME	Tumor immune microenvironment
Tregs	Regulatory T cells
Trp	Tryptophan
TME	tumor microenvironment
